# Identification, Characterization and Antibiotic Resistance of Bacterial Isolates Obtained from Waterpipe Device Hoses

**DOI:** 10.3390/ijerph120505108

**Published:** 2015-05-13

**Authors:** Majed M. Masadeh, Emad I. Hussein, Karem H. Alzoubi, Omar Khabour, Muhamad Ali K. Shakhatreh, Mahmoud Gharaibeh

**Affiliations:** 1 Department of Pharmaceutical Technology, Jordan University of Science and Technology, Irbid 22110, Jordan; 2 Department of Biology, Yarmouk University, Irbid 22110, Jordan; E-Mail: Shussein5@yu.edu.jo; 3 Department of Clinical Pharmacy, Jordan University of Science and Technology, Irbid 22110, Jordan; E-Mail: khalzoubi@just.edu.jo; 4 Department of Medical Laboratory Sciences, Faculty of Applied Medical Sciences, Jordan University of Science and Technology, Irbid 22110, Jordan: E-Mails: khabour@just.edu.jo (O.K.); mkshakhatreh@just.edu.jo (M.A.K.S.); 5 Department of Biology, Faculty of Science, Taibah University, Medina 41411, Saudi Arabia; 6 Princesses Basma Teaching Hospital, Irbid 21110, Jordan; E-Mail: mahmoudgharaibeh718@yahoo.com

**Keywords:** waterpipe, hose, bacteria, isolates, antibiotics resistance

## Abstract

The general lack of knowledge about the health effects of waterpipe smoking is among the reasons for its global spread. In this study, bacterial contamination of waterpipe hoses was investigated. Twenty hoses were collected from waterpipe cafés and screened for bacterial pathogens using standard culture and isolation techniques. Additionally, resistance of isolated bacteria to common antibiotics was determined by identifying the minimum inhibitory concentration (MIC) of each isolate. Forty eight bacterial isolates were detected. Isolates included both Gram-positive and Gram-negative pathogens from species that included *Micrococcus* (12), *Corynebacterium* (13) and *Bacillus* (9). In addition, some of the detected pathogens were found to be resistant to aztreonam (79%), cefixime (79%), norfloxacin, amoxicillin (47%), clarithromycin (46%) and enrofloxacin (38%). In conclusion, the hose of the waterpipe device is a good environment for the growth of bacterial pathogens, which can then be transmitted to users.

## 1. Introduction

Waterpipe is a general term used to describe a mode of tobacco consumption where the smoke produced by burning the tobacco passes through water before it is inhaled by the users. Waterpipe tobacco smoking is increasing in popularity worldwide, especially among young users and women. In some countries, the number of waterpipe smokers exceeds the number of cigarette users [1,2,3]. Reasons behind the increase in waterpipe use are the belief that the harmful substances in the tobacco is filtered by the water in the bowl [4] and the general lack of knowledge regarding the health effects of waterpipe tobacco smoking.

**Figure 1:**
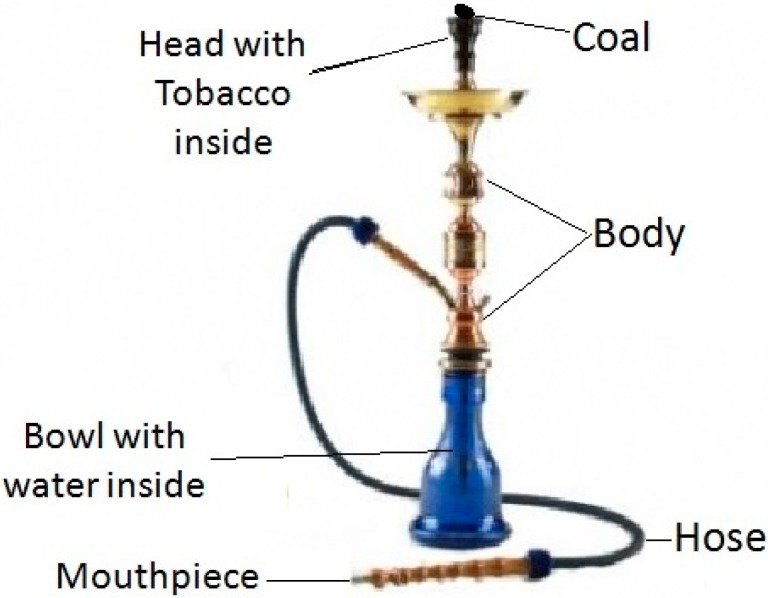
The structure of a waterpipe device.

The common structure of the device that used for waterpipe tobacco smoking is shown in Figure 1. The moist sweetened tobacco (called Mo’assal) is placed in the head and is burned by lit charcoal. The mainstream smoke aerosol is produced when the smoker sucks through the hose via the mouthpiece [5,6].

A previous study has shown the presence of microbial and fungal biomarkers in waterpipe smoke, which they termed “bioaerosol” [7]. The most frequent contaminants of the waterpipe bowl and the mouth pieces were *Staphylococci*, *Streptococcus* spp, *Neisseria* spp and *Escherichia coli* [8] and it was shown that waterpipe use was associated with a higher incidence of respiratory illnesses [9].

Since users require a waterpipe device to smoke, they usually smoke at their family or friends’ homes. In addition, they smoke in public sites where the device is available such as cafés (hooka bars), restaurants and picnic areas [5]. The device including the hose is usually used by multiple individuals and the only part that gets changed is the mouthpiece [3]. Thus, a valid health concern is that waterpipe can transmit pathogens including bacteria and viruses through the shared hose of the device. In this study, types of bacteria presents in waterpipe hoses in waterpipe cafés were identified via the collection of random hose swab samples in the city of Irbid (Jordan). In addition, resistance profiles of detected bacterial isolates to common antibiotics were also determined.

## 2. Experimental Section

### 2.1. Bacterial Isolation

In this cross sectional study, twenty waterpipe hoses were randomly selected from five waterpipe cafés (hooka bars that mostly provide waterpipes in addition to serving hot or cold non-alcoholic drinks) out of 30 located in Irbid, which is the second largest urban area located in the north of Jordan. The selection was based on geographical distribution of cafés (north, south, east, and west, and downtown). Four samples were selected from each café via a random draw of a table number/occupied seat number. The base part of the waterpipe hose (about 10 cm from the waterpipe body) was swabbed. Swabs were inoculated on nutrient agar (N.A) and Triptone Soya Agar (TSA) media and incubated for 48 h under aerobic conditions. After incubation bacterial colonies were selected according to variation in morphological characteristics (size, shape, color, and margin). The selected bacterial colonies were transferred into fresh N.A and TSA media to insure purity of isolates.

### 2.2. Biochemical and Physiological Characterization

A series of biochemical tests and selective media, which include MacConkey agar, *Pseudomonas* agar, Simmons Citrate agar, eosin methylene blue agar (EMB) and *Salmonella* agar were used to characterize each bacterial isolate. Isolated strains were characterized using standard microbiological methods as described in Clinical and Laboratory Standards Institute (CLSI) ML35-A2 document [10]. The following tests were used: catalase test, oxidase test, nitrate reduction test, methyl red test, Voges-Proskauer test, indole production test, HL media (O/F), urea, gelatin, gas and acid production from D-lactose, D-galactose, D-sucrose, D-arabinose, D-maltose, D-fructose and D-mannitol, utilization of citrate test, blood hemolysis test and triple sugar iron test [10].

### 2.3. Antimicrobial Screening of Selected Antibiotics: Disc Diffusion Assay

This assay was employed to determine the antimicrobial activity of selected antibiotics against the identified bacterial strains. The method used was carried out as described previously [11,12]. A concentration of 100 mg/mL of each antibiotic was prepared. Overnight log phase that corresponds McFarland’s standard 0.5 cultures were used for the screening. A cotton swab was used to streak the bacteria on Mueller Hinton agar to form a bacterial lawn. The discs were placed on the agar using a sterile forceps. The plates were left to dry at 4 °C for 1 h. Plates were then incubated for 24 h at 37 °C. Distilled water was used as the negative control. The zone of inhibition was measured in millimeters and recorded. Minimum inhibitory concentration (MIC): MIC is the lowest concentration of drug that inhibits the growth of bacteria. It is the concentration that gives the least inhibitory mode of action and below which there is no further inhibition. For this test, a 24-well microtitre plate was used. Precisely, 1 mL of LB broth was placed in all wells. Antibiotic stock (1 mL) was placed in the well labeled “A”. A two-fold serial dilution was performed. The mixtures were resuspended with a pipette each time before they were transferred to the next well. A 100 µL of bacteria was inoculated in all wells. Distilled water was used as the negative control. The plates were incubated at 37 °C for 24 h. The wells were then observed for turbidity or any sign of growth.

### 2.4. Data Processing and Analysis

Collected data were keyed into Microsoft Excel 2010, and presented as bar charts or as frequency tables with number and percentages within each column for categorical data. For continuous data (the MIC), average values of at least three experiments were presented for each bacterial strain.

## 3. Results and Discussion

**Figure 2:**
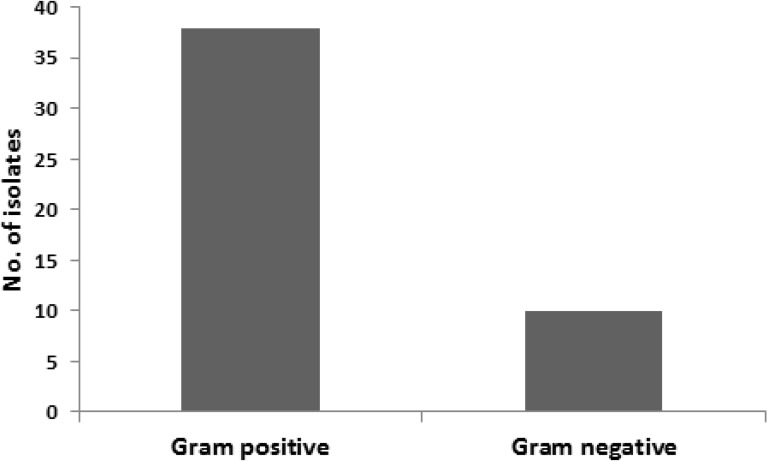
Distribution of bacterial isolates based on Gram stain.

**Figure 3:**
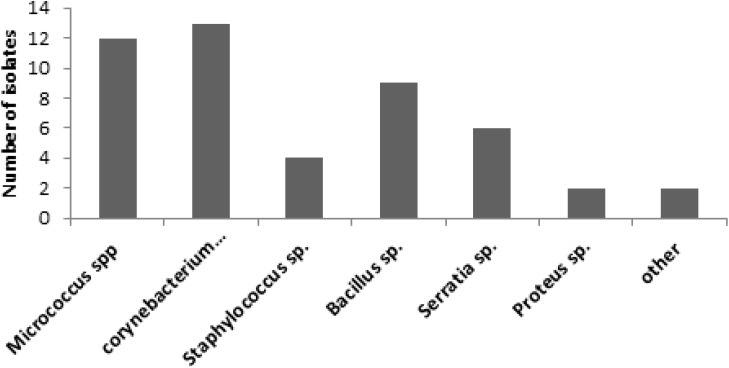
Distribution of bacterial isolates from collected hoses.

Forty eight bacterial isolates were obtained from the twenty waterpipe hoses. The majority of bacterial isolates were Gram positive bacteria (Figure 2) belonging to the *Micrococcus* (*n =* 12), *Corynebacterium* (*n =* 13) and *Bacillus* (*n =* 9) species (Figure 3).

Bacterial isolates were examined for their susceptibility to common antibiotics (Table 1). *Microccocus*, *Proteus vulgaris* and *Bacillus subtilis* isolates showed high sensitivity to norfloxacin while they were less susceptible to cephalexin. *Sarratia fonticola*, *Bacillus cereus* isolates were highly sensitive to tetracyclin, while they were less susceptible to cephalxin*. Pasedo diphtheriticum* was relatively less sensitive to amoxicillin while *Staphylococcus aureus* was relatively less sensitive to cefixime. The frequencies of resistance of isolated strains to common antibiotic are shown in Table 2.

**Table 1:** MIC (mg/mL) of different antibiotics against selected bacterial species (values are average of at least three experiments).

Bacterial Species	Norfloxacin (mg/mL)	Amoxicillin (mg/mL)	Cefixime (mg/mL)	Tetracycline (mg/mL)	Cephalexin (mg/mL)
*Microccoccus*	0.048	0.6	2.56	0.08	6.4
*Proteus vulgaris*	0.048	1.2	0.16	0.08	6.4
*Serratia fonticola*	0.384	0.3	0.64	0.08	6.4
*Pasedo diphtheriticum*	0.192	1.2	0.64	0.08	0.1
*Bacillus cereus*	0.384	3.2	1.28	0.06	3.2
*Bacillus subtilis*	0.024	0.6	2.56	0.8	6.4
*Staphylococcus aureus*	0.048	0.6	1.28	0.2	0.08
*Listeria monocytogenes*	0.024	0.6	0.32	0.08	0.06

**Table 2:** Antibiotic sensitivity of bacterial isolates.

Antibiotic	Number of Resistant Isolates	Frequency of Resistant Isolates (out of 48)
Aztreonam	38	79%
Azithromycin	13	27%
Amoxicillin	23	47%
Cefixime	38	79%
Imipenem	10	21%
Trimethoprim/sulfamethoxazole	11	23%
Piperacillin/tazobactam	9	19%
Clarithromycin	22	46%
Enrofloxacin baytril	18	38%
Norfloxacin	6	13%

In the current study, we characterized bacteria present in waterpipe hoses. The results showed the presence of a wide spectrum of Gram-positive and Gram-negative strains. In addition, bacterial isolates showed variations in their susceptibility to major antibiotics.

The lack of knowledge regarding the health effects of waterpipe tobacco smoking is suggested to be one reason for its global spread. It is believed that the water in waterpipe device bowl filters the smoke rendering it less harmful than other types of smoking [4]. Previous literature has shown that the smoke inhaled by a waterpipe smoker contains a profile of toxicant compounds comparable to that found in cigarette smoke [13]. Such toxicants include carcinogenic polycyclic aromatic hydrocarbons [14], carbon monoxide, heavy metals, aldehydes and others [15]. In addition, waterpipe tobacco smoking has been shown to increase DNA damage in the lymphocytes and buccal mucosa cells of the users [16,17,18,19]. It also has been shown to be associated with lung cancer [9,20]. More recently, waterpipe tobacco smoking has been shown to interfere with respiratory and vascular functions [21,22]; and oral diseases [23,24].

The results presented in this study indicate the presence of bacterial pathogens in the hoses of waterpipe devices. In addition, some of these pathogens showed significant levels of resistance to antibiotics. Recently, Markowicz *et al.* [7], reported the presence of microbial (*viz* 3-hydroxy fatty acids) and fungal (ergosterol) biomarkers in waterpipe smoke, which they termed “bioaerosol”. In addition, *Staphylococci*, *Streptococcus* spp, *Neisseria* spp and *Escherichia coli* have been shown to be frequent contaminants of the water bowl and mouthpiece of the waterpipe device [8]. Since waterpipe smoking is seen by most of the users as a social activity and sharing the same device is common behavior, the data presented in this study and those of others [7,8] suggest that waterpipe use is associated with high risk of transmission of infectious diseases.

That data also indicates the need for regulation of waterpipe products that include the device itself. In addition, since waterpipe devices in the cafes are used by several individuals in the same day, regulations regarding cleaning the devices including hoses are essential to limit transmission of pathogens. One of the limitation of the current study is the relatively low number of hoses (*n =* 20) that were examined. In addition, the smoking behavior, design of waterpipe instruments and cleaning procedures might vary among countries. Therefore, the data presented in the current study need to be confirmed by a lager study that include more hoses and from different countries. Additionally, laws must be in place to limit the public use/sharing of waterpipe devices.

## 4. Conclusions

The hoses of waterpipe devices might be a good environment for the growth of bacterial pathogens that can be transmitted to users, and that show resistance to commonly used antibiotics, thus they could be the source for potential infections outbreaks.
